# Pancreaticoduodenectomy with malrotation following reoperation due to torsion of efferent loop: a case report

**DOI:** 10.1186/s40792-020-00991-x

**Published:** 2020-09-25

**Authors:** Yumiko Kageyama, Ryuzo Yamaguchi, Shinya Watanabe, Keiji Aizu, Shinichiro Kobayashi, Fumiya Sato, Hironori Fujieda, Yoshitaka Toyoda, Tsutomu Iwata

**Affiliations:** Department of Surgery, Kasugai Municipal Hospital, 1-1-1 Takaki-cho, Kasugai-shi, Aichi Japan

**Keywords:** Pancreaticoduodenectomy, Malrotation, Reconstruction, Reoperation

## Abstract

**Background:**

Malrotation is a congenital anomaly during the development of the embryonic intestine. Although it is generally considered a pediatric surgical condition, it can have significant implications for adult surgery in terms of reconstruction.

**Case presentation:**

The patient was an 85-year-old man with pancreatic cancer and intestinal malrotation. He underwent pancreaticoduodenectomy with modified Child’s reconstruction. Because the ascending colon and efferent loop twisted easily, we fixed the ascending colon to the abdominal wall. Thereafter, right twist and stenosis of the efferent loop occurred. On the 22nd day after the initial surgery, detorsion and Braun anastomosis were performed for efferent loop fixation. Postoperative oral intake was good, and the patient was discharged from our hospital on the 24th day after the reoperation.

**Conclusions:**

This is a rare case of pancreaticoduodenectomy with malrotation following reoperation due to a complication after Child’s reconstruction. In similar cases of intestinal malrotation, it is important to consider avoiding coaxial positioning of intestinal parts and an upper abdominal space while selecting a reconstruction method.

## Background

Intestinal malrotation is a developmental anomaly of the embryonic intestine, which is frequently observed in neonates. Although it occurs rarely in adults, it influences the condition of the intestines and has significant implications for reconstruction surgery. Here, we present the case of a patient who underwent pancreaticoduodenectomy and had intestinal malrotation following reoperation due to torsion of the efferent loop.

## Case presentation

**Fig. 1:**
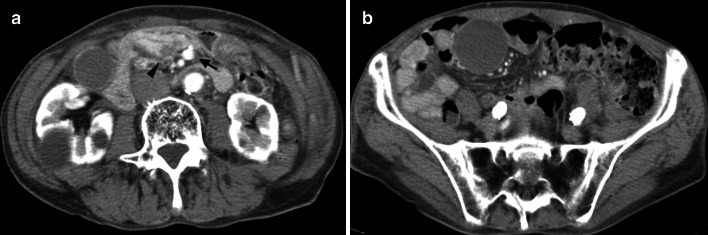
Computed tomography findings. **a** A 25-mm low-density mass located in the pancreatic head (arrowhead). The superior mesenteric vein (SMV) was located to the left of the superior mesenteric artery, showing the SMV rotation sign (arrow). **b** The small bowel was located in the right abdomen, and the large bowel was located in the left abdomen

**Fig. 2:**
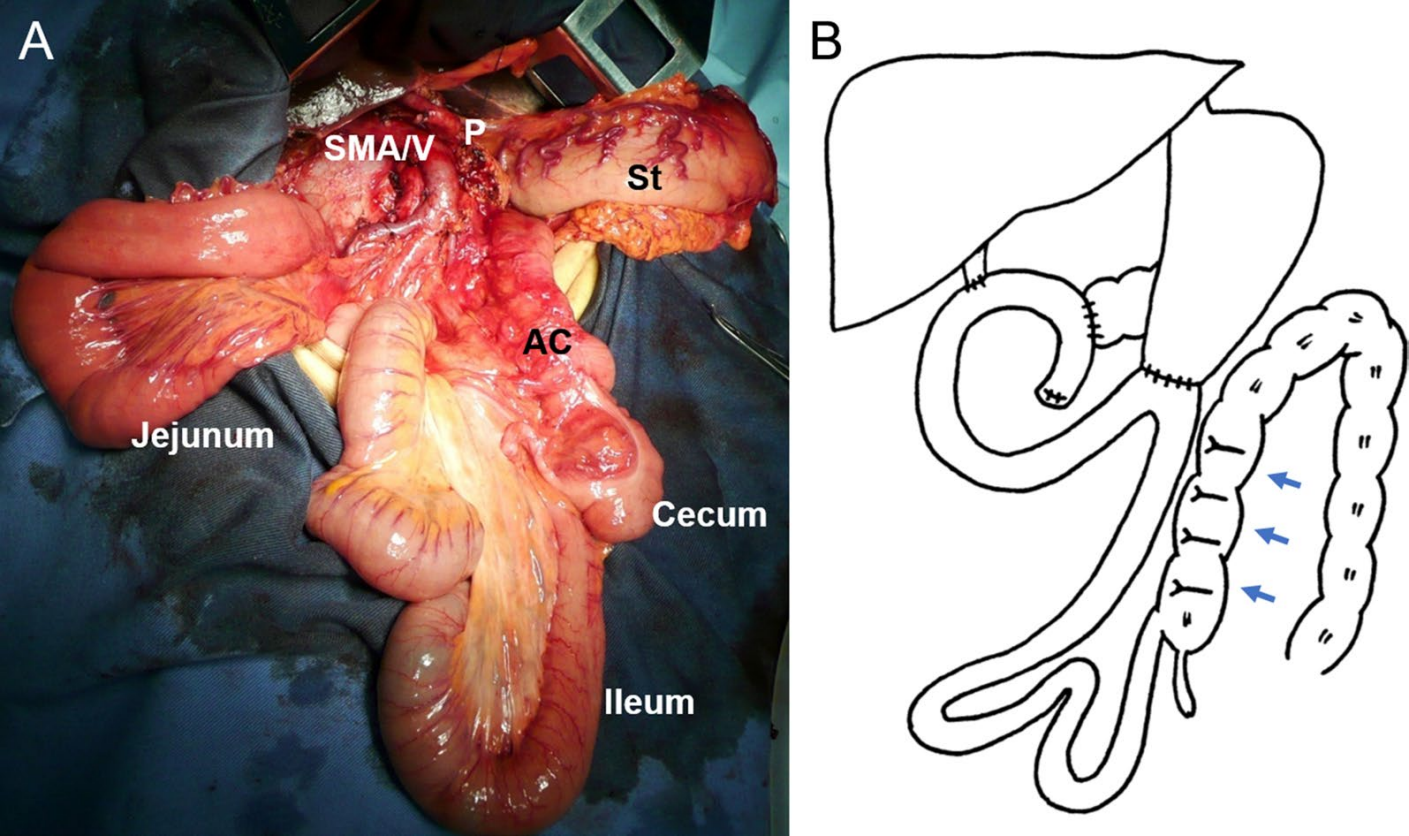
Findings in the initial operation. **a** The ligament of Treitz was absent, and the small bowel was in the right abdomen. Further, the ascending colon and cecum were not fixed to the retroperitoneum but were located in the median abdomen. AC, ascending colon; P, pancreas; St, stomach; SMA/V, superior mesenteric artery and superior mesenteric vein. **b** Pancreaticoduodenectomy with reconstruction using the modified Child’s method was performed. We fixed the ascending colon to the abdominal wall because the ascending colon and efferent loop twisted easily (arrow)

The patient was an 85-year-old man who presented with appetite loss and choluria. His past medical history included hypertension, tuberculosis, and ulcerative colitis. Laboratory examination revealed elevated hepatobiliary enzyme and serum tumor marker levels: total bilirubin, 21.2 (normal range, 0.1–1.2) mg/dL; aspartate aminotransferase, 83 (normal range, 5–30) IU/L; alanine aminotransferase, 103 (normal range, 3–35) IU/L; alkaline phosphatase, 1699 (normal range, 90–300) IU/L; γ-glutamyl transpeptidase, 926 (normal range, 1–28) IU/L, carcinoembryonic antigen, 5.9 (normal range, 0–5) ng/mL; carbohydrate antigen 19–9, 1966 (normal range, 0–37) ng/mL, and s-pancreas-1 antigen, 371.5 (normal range, 0–30) U/mL. Computed tomography revealed a low-density pancreatic head mass (diameter: 25 mm) and dilation of the common bile duct and pancreatic duct. The superior mesenteric vein (SMV) was located to the left of the superior mesenteric artery, and this phenomenon is known as the SMV rotation sign (Fig. 1a). The duodenal sweep did not cross the midline, the small bowel was located in the right abdomen, and the large bowel was located in the left abdomen (Fig. 1b). These findings are consistent with the characteristics of malrotation. Endoscopic retrograde cholangiopancreatography revealed interruptions in the continuity of the common bile duct and main pancreatic duct, and brush cytology showed pseudo-positive findings. Based on these findings, the patient was diagnosed with pancreatic cancer accompanied by malrotation. During laparotomy, we noticed that the ligament of Treitz was absent, the small bowel was in the right abdomen, and the ascending colon and cecum were not fixed to the retroperitoneum but were located in the median abdomen (Fig. 2a). These findings indicated the nonrotation-type malrotation. Pancreaticoduodenectomy with reconstruction using the modified Child’s method was performed. In addition, we fixed the ascending colon to the abdominal wall because the ascending colon and efferent loop twisted easily (Fig. 2b). The pathological diagnosis was as follows: tubular adenocarcinoma, moderately differentiated, and pT2N1M0 Stage IIB in accordance with the eighth edition of the UICC TNM Classification.

**Fig. 3:**
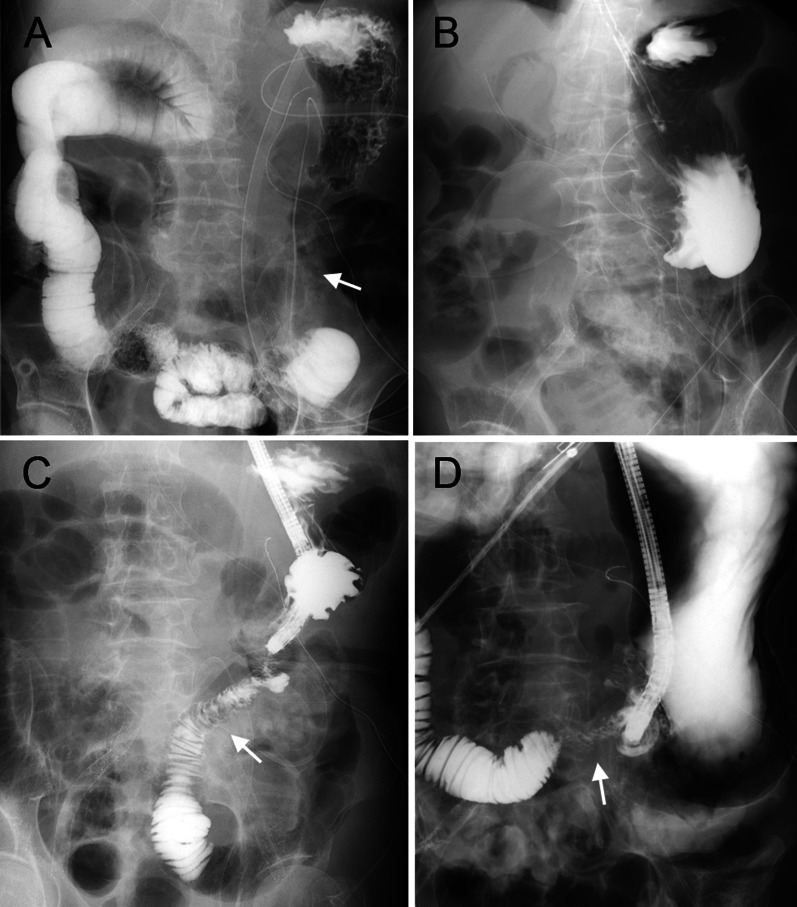
Findings of upper gastrointestinal series after the initial operation. **a **A contrast medium easily flowed into the efferent loop (arrow) on postoperative day (POD) 4*.* **b** Contrast medium did not flow into the efferent loop on POD8. **c** Although upper endoscopy revealed anastomotic edema and stricture, contrast medium flowed into the efferent loop (arrow) on POD12. **d** The efferent loop (arrow) was twisted rightward on POD20

**Fig. 4:**
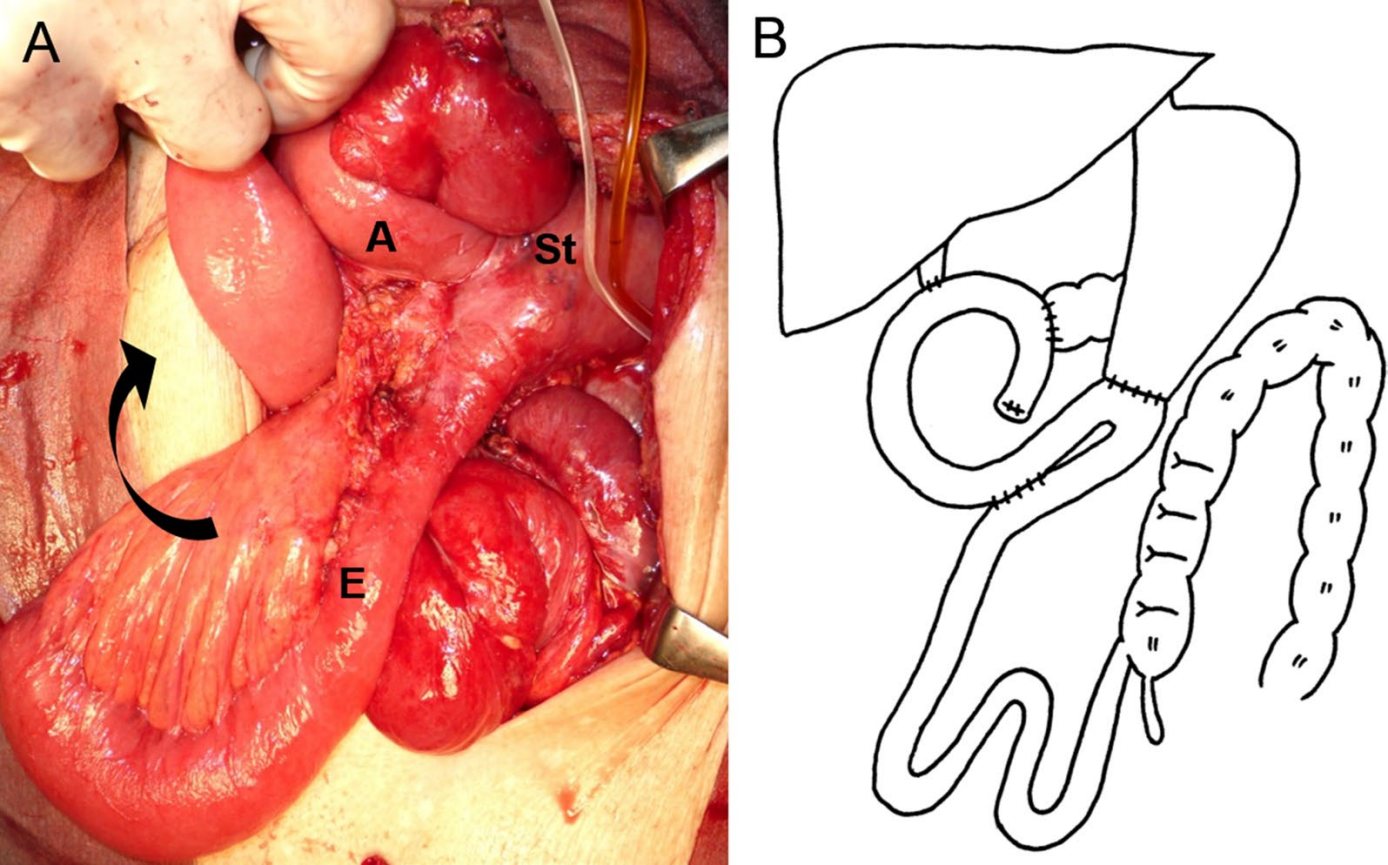
Findings of the reoperation. **a** After detorsion, the efferent loop had twisted 180° in the clockwise direction and was adherent to the right upper abdominal wall (black arrow). **b** Braun anastomosis was performed to prevent efferent loop twisting and maintain its continuity inferiorly. A, afferent loop; E, efferent loop; St, stomach

On postoperative 4th day, upper gastrointestinal (UGI) series showed that contrast medium easily flowed through the intestines (Fig. 3a), and the patient was prescribed a postoperative diet. On postoperative 8th day, the patient vomited and UGI series showed that contrast medium did not flow into the efferent loop (Fig. 3b). On postoperative 12th day, upper endoscopy revealed anastomotic edema and stricture; however, contrast medium flowed into the efferent loop (Fig. 3c). Although we treated the patient conservatively, he did not respond to the treatment. On postoperative 20th day, the upper endoscope could not pass through the anastomotic site and UGI series revealed right efferent loop twisting (Fig. 3d). On postoperative 22nd day, reoperation was performed. During laparotomy, it was found that the efferent loop had twisted 180° in the clockwise direction and was adherent to the right upper abdominal wall (Fig. 4a). Detorsion and Braun anastomosis were performed to prevent torsion and maintain the efferent loop continuity inferiorly (Fig. 4b). Postoperative oral intake was good, and the patient was discharged from our hospital on the 24th day after reoperation.

## Discussion

Intestinal malrotation is a congenital anomaly, with 80% of cases occurring in neonates [1]. The remaining cases are not diagnosed until adulthood and often show asymptomatic incidental findings [2].

Wang et al. [3] classified intestinal malrotation cases into four types: (1) nonrotation, (2) malrotation, (3) reversed rotation, and (4) paraduodenal. Accordingly, our case was diagnosed as nonrotation-type intestinal malrotation based on 90° rotation in the counter clockwise direction.

When performing pancreaticoduodenectomy in patients with malrotation, factors such as intestine position, vascular anomalies, and reconstruction procedures should be considered. The Ladd procedure has traditionally been performed to treat malrotation, and it entails division of Ladd’s bands, detorsion of the volvulus, and reorientation of the small bowel in the right intestine and the colon in the left intestine [4]. In cases associated with malignancy, it is important to choose an appropriate reconstruction procedure in addition to treatment for intestinal malrotation. However, no report on the reconstruction procedure was found. We conducted a review of literature that included Japanese studies by searching the PubMed database, and we found reports on a total of 16 patients with intestinal malrotation who underwent pancreaticoduodenectomy (Table 1) [5–19]. None of the 16 patients developed any complication associated with reconstruction.

**Table 1:** Reported cases of pancreaticoduodenectomy with intestinal malrotation

Author/Year	Age/sex	Primary disease	Type	Reconstruction method	Braun anastomosis	Ladd’s operation	intestinal fixation
Jagannath [5] 1995	59 M	Pancreatic cancer	Reversed rotation	Whipple	−	−	−
Sato [6] 1997	44 F	Duodenal adenoma	Nonrotation	n.a	n.a	+	n.a
Mateo [7] 2005	71 M	Pancreatitis	Nonrotation	Traverso	n.a	+	n.a
	43 M	Ampullary cancer	Nonrotation	n.a	n.a	−	−
Hayashi [8] 2010	61 M	Bile duct cancer	Nonrotation	n.a	n.a	−	n.a
Plackett [9] 2011	69 F	Pancreatic cancer	Nonrotation	Child	−	+	n.a
Owada [10] 2012	54 M	Pancreatic cancer	Nonrotation	Child	+	+	−
Kawahara [11] 2013	63 M	Ampullary cancer	Incomplete fixation	n.a	n.a	−	n.a
Lim [12] 2014	59 M	Bile duct cancer	Atypical rotation	Child	n.a	−	n.a
Saito [13] 2015	74 M	Bile duct cancer	Nonrotation	Child	n.a	−	n.a
Tsutsumi [14] 2016	54 M	Pancreatic cancer	Reversed rotation	Child	+	−	−
Takishita [15] 2017	78 M	Bile duct cancer	n.a	Child	n.a	n.a	−
Yagi [16] 2017	72 F	Ampullary cancer	n.a	Child	−	n.a	−
Yamashita [17] 2018	75 F	Ampullary cancer	Nonrotation	Child	n.a	−	−
Li [18] 2018	76 M	Ampullary cancer	n.a	Whipple	n.a	n.a	−
Tanaka [19] 2018	61 M	Ampullary cancer	Nonrotation	Child	+	+	−
Our case	85 M	Pancreatic cancer	Nonrotation	Child	−	−	+

*n.a.* not available

Kawano et al. [20] reported that a patient with intestinal malrotation required reoperation for reflux esophagitis followed by total gastrectomy with Billroth II reconstruction. In their patient, the lifted jejunum released into the left upper abdomen because the intestines were not adequately fixed to the retroperitoneum. Roux-en-Y anastomosis and intestinal fixation were performed during the reoperation, and the necessity of intestinal fixation was explained. In contrast, Stauffer et al. [21] reported that additional fixation was unnecessary.

There were two complications of child’s reconstruction in our patient. First, there was a risk of detorsion of the volvulus given that the ascending colon was adjacent and positioned coaxially to the efferent loop. We prevented its torsion by additionally fixing the ascending colon to the abdominal wall. However, reconstruction may best be performed using the Cattell’s approach or Imanaga’s method, particularly in cases such as our case, because the ascending colon cannot be positioned coaxially to the efferent loop in these methods. Second, there was a space in the right upper abdomen that the efferent loop easily entered. We usually do not perform Braun anastomosis along with pancreaticoduodenectomy because we believe that Braun anastomosis is not necessary in all cases. In the present case, although Braun anastomosis was useful in the stabilization of the efferent loop, the large space in the upper abdomen could have been avoided if reconstruction was performed using Cattell’s approach or Imanaga’s method. These methods might be useful in the cases of nonrotation-type intestinal malrotation or some cases of reversed rotation-type intestinal malrotation; however, a definite recommendation cannot be given considering the different types of anomalies. In addition, Braun anastomosis or intestinal fixation should be considered.

## Conclusions

We presented a rare case of pancreaticoduodenectomy with malrotation following reoperation due to a complication of a surgical method. Coaxial positioning of intestinal parts and an upper abdominal space should be avoided in patients with intestinal malrotation who undergo gastrointestinal tract reconstruction.

## Data Availability

The data will not be shared, because there is no available data to be shared.

## Abbreviations

SMV: Superior mesenteric vein
UGI series: Upper gastrointestinal series

## References

[1] Snyder WH, Chaffin L (1954). Embryology and pathology of the intestinal tract: presentation of 40 cases of malrotation. Ann Surg.

[2] Gohl ML, DeMeester TR (1975). Midgut nonrotation in adults. An aggressive approach. Am J Surg.

[3] Wang CA, Welch CE (1963). Anomalies of intestinal rotation in adolescents and adults. Surgery.

[4] Ladd WE (1936). Surgical diseases of the alimentary tract in infants. N Engl J Med.

[5] Jagannath P, Albuquerque K, de Souza LJ, Mohandas KM, Shantiswaroop V (1995). Reversed rotation of midgut which caused problems during Whipple's procedure. Eur J Surg.

[6] Sato T, Mochizuki T, Tanaka T, Okazaki T, Sanada Y (1997). A case of duodenal tumor with intussusception and intestinal malrotation. J Jpn Surg Assoc.

[7] Mateo R, Stapfer M, Singh G, Sher L, Jabbour N, Selby RR (2005). Pancreaticoduodenectomy in adults with congenital intestinal rotation disorders. Pancreas.

[8] Hayashi T, Takano S, Kimura F, Shimizu H, Yoshidome H, Ohtsuka M (2010). A case of cholangiocarcinoma with hepatomesenteric trunk and intestinal malrotation treated with pancreaticoduodenectomy. Jpn J Cancer Chemother.

[9] Plackett TP, Takamori R, Izawa M (2011). Pancreaticoduodenectomy in the setting of intestinal malrotation. Hawaii Med J.

[10] Owada Y, Matsuo R, Ikeda O, Nakayama K, Tanoi T, Ohkohchi N (2012). Case of pancreatic cancer with intestinal malrotation treated by pancreaticoduodenectomy. J Jpn Surg Assoc.

[11] Kawahara R, Horiuchi H, Nogita H, Akashi M, Mikagi K, Yoshitomi M (2013). A case of cancer of the ampulla of Vater accompanied by malrotation. Kurume Med J.

[12] Lim HK, Choi YS, Lee SE, Kang H (2014). Pancreaticoduodenectomy performed in a patient with situs ambiguous accompanied with isolated levocardia, malrotation, and normal spleen. Ann Surg Treat Res.

[13] Saito Y, Miyamoto A, Maeda S, Hama N, Haraguchi N, Yamamoto K (2015). A case of cholangiocarcinoma with intestinal malrotation treated with pancreaticoduodenectomy. Jpn J Cancer Chemother.

[14] Tsutsumi S, Toyoki Y, Kagiya T, Kimura T, Kimura N, Kudo D (2016). Pancreatic head cancer with adult reversed rotation treated by pancreaticoduodenectomy. Jpn J Gastroenterol Surg.

[15] Takishita C, Nagakawa Y, Hosokawa Y, Sahara Y, Katsumata K, Tsuchida A (2017). A case of distal bile duct cancer associated with preduodenal portal vein, preduodenal common bile duct, and intestinal malrotation. J Jpn Surg Assoc.

[16] Yagi N, Arakawa K, Andoh T, Tomizawa N, Shimizu H, Honda R (2017). A case report of duodenal papillary carcinoma with situs inversus totalis and anomalies treated with pancreatico-duodenectomy. J Clin Surg.

[17] Yamashita S, Hiroyoshi J, Hasegawa K (2018). Pancreatoduodenectomy for patients with intestinal malrotation. Geka.

[18] Li WT, Sethi S, Christopher AN, Koganti D, Yeo CJ (2018). Duodenal adenocarcinoma in a patient with partial intestinal malrotation. J Pancreat Cancer.

[19] Tanaka H, Suzuki Y, Kawarada Y, Kitashiro S, Okushiba S, Hirano S (2018). A case of ampullary cancer with adult intestinal malrotation treated by pancreaticoduodenectomy. J Jpn Surg Assoc.

[20] Kawano F, Sekiya R, Shinohara T, Uchino H, Onitsuka T (2006). A case of gastric cancer with malrotation of intestine required re-operation for reflex esophagitis after total gastrectomy. J Jpn Surg Assoc.

[21] Stauffer UG, Herrmann P (1980). Comparison of late results in patients with corrected intestinal malrotation with and without fixation of the mesentery. J Pediatr Surg.

